# Editorial: Metabolic responses and adaptations to exercise

**DOI:** 10.3389/fspor.2024.1416649

**Published:** 2024-05-08

**Authors:** Tania Gamberi, Cristina Vassalle, Pantelis Theodoros Nikolaidis, Alessio Pellegrino, Simone Luti

**Affiliations:** ^1^ Department of Experimental and Clinical Biomedical Sciences, School of Mathematical, Physical and Natural Sciences, University of Florence, Florence, Italy; ^2^ Department of Laboratory Medicine, Gabriele Monasterio Tuscany Foundation (CNR), Pisa, Italy; ^3^ School of Health and Welfare Sciences, University of West Attica, Athens, Greece; ^4^ Department of Experimental and Clinical Medicine, University of Florence, Florence, Italy

**Keywords:** acute exercise, chronic exercise, sport metabolism, healthy aging, performance

**Editorial on the Research Topic** Metabolic responses and adaptations to exercise

Sedentary lifestyle is one of the main problems of modern society and is responsible for the dramatic growth of overweight and obesity, which are also powerful risk factors for serious chronic diseases. Conversely, physical exercise is proposed as a highly effective tool of treating and preventing the main causes of morbidity and mortality—associated with aging—in industrialized countries. Numerous epidemiological and prospective studies have reported a strong association between physical activity and morbidity–mortality index of population, even in overweight and obese person.

In fact, several metabolic changes occur in the organism during exercise, leading to the activation of adaptive mechanisms. These mechanisms aim to establish a new dynamic equilibrium especially at the metabolic level, which enhances health and optimize performance in elite athletes.

Clearly, many variables (e.g., exercise mode, frequency, duration, and intensity) may affect the results.

What molecular mechanisms are activated in response to physical exercise? And in recovery, do they act similarly? How regular physical exercise is associate with health, especially in elderly people? What benefits arise from physical activity as an expression and activation of molecular mechanisms?

The present Research Topic aimed to address the above-mentioned research questions including ten scientific papers.

Chmielecki et al. examined the effect of an exhaustive run on Interleukin IL-4, IL-8, IL-10, and Tumor Necrosis Factor *α* concentrations in 16 amateur athletes, and observed that, although IL-4 did not change after exercise, its baseline value negatively correlated with post-exercise luminescence. Based on these findings, the researchers concluded that plasma IL-4 might be related to preservation of an optimal balance between oxidants and antioxidants during and after exercise.

Abdalla-Silva et al. investigated the role of the β2-Adrenoceptors (β2-ARs) blockade on the acute molecular responses induced by a single session of resistance exercise in rodent skeletal muscles. Considering their findings, the researchers suggested that β2-ARs stimulation during acute resistance exercise stimulated the hypertrophic gene Nr4a3 and inhibited the overexpression of atrophic genes in the first hours of postexercise recovery, highlighting the impact of the sympathetic nervous system on muscle adaptations.

In another study, Meihua et al. studied sweat samples using chemical isotope labeling liquid chromatography-mass spectrometry before and after high-intensity interval exercise-induced fatigue in 14 long-distance runners and identified 446 metabolites and the sweat metabolome group. They concluded that alterations of hypoxanthine concentration in sweat might be used as a biomarker for the diagnosis of exercise-induced fatigue, whereas the change of pyruvate concentration in sweat might be used as a discriminant index for the energy metabolism mode of the body pre- and post-exercise.

Ye et al. studied the impact of acute exercise on the metabolome and transcriptome profiles in mice skeletal muscle, by using an integrative and holistic approach combining multi-omics technologies. Results revealed the complex network between metabolites and genes, identifying 34 differentially expressed metabolites (28 up-regulated and 6 down-regulated), as well as 245 differentially expressed genes (155 up-regulated and 90 down-regulated genes), involved in different metabolic and signaling pathways related to the exercise-induced physiological regulation of skeletal muscle. These data help to better understand the molecular changes that follow exercise in skeletal muscle using omics technologies, evidencing innovative potential future therapeutic targets related with exercise.

Jia et al. discussed the role of exerkines in osteoarthritis, some with beneficial other with adverse effects in the disease (e.g., irisin, lactate, secreted frizzled-related protein, neuregulin, and adiponectin favourable, while IL-6, IL-7, IL-15, IL-33, myostatin, fractalkine, follistatin-like 1, visfatin, activin A, migration inhibitory factor, apelin and growth differentiation factor-15 with aggravating effects). These exerkines can be modulated by exercise with effects on the disease (although some type or load in terms of intensity and frequency of exercise can elicit adverse exerkines as well) and can serve as new promising pharmacological targets to develop future personalized interventional strategies.

Wang et al. explored the effects and underlying mechanisms of exercise in treating lumbar disc herniation (characterised by lumbar disc degeneration, annulus fibrosus rupture, herniation of the nucleus pulposus irritate and nerve compression, resulting in lumbar and/or lower extremity pain). Three main mechanisms by which exercise appears to exert its benefits were discussed: mechanical compression, inflammatory chemical stimulation, and autoimmunity, whereas the use of non-acute self-weighted exercise types, exercise durations exceeding two weeks, and non-high-intensity exercise therapies appear, in view of actual available evidence, more effective to reduce disease severity and alleviate symptoms.

Meloni et al. measured fat oxidation rates and cardiorespiratory responses during exercise in trained and recreational athletes with post-acute sequelae of SARS-CoV2-infection; results, although requiring further confirmation in additive future studies, suggested that specific endurance training, when compared with a simply active lifestyle, might be more protective against alterations caused by virus infection, such as mitochondrial dysfunction or, more in general, abnormalities of the fatty acid oxidation pathway.

Qi et al. summarized the progress in the application of metabolomics in sport science. They discussed the advantage and disadvantage of the different chemical analysis platform and data analysis. In this review the authors pointed out the role of metabolomics in searching for potential biomarkers and therapeutic targets by detecting metabolite changes in a variety of biological fluids and tissues.

Majed et al. studied the metabolic, perceptual, spatiotemporal and gait stability parameters of walking around the preferred speed (PWS) in thirty-four healthy and sedentary volunteers (18 women and 16 men) aged between 18 and 26 years. Main findings indicated the increase of fat oxidation suggesting benefits of walking at the preferred walking speed for sedentary young adults compared to walking at a slower or faster intensity.

Ryningen et al. analyzed the expression levels of circulating microRNAs in elite cyclists after heavy strength (HS) and short-sprint (SS) training methods with the aim to find potential biomarkers for individual optimal restitution time. They found that both training methods can increase the circulating levels of some of the miRNAs associated with muscle development such as myomiRs miR-1-3p, 133a-3p and 133b-3p suggesting their relevance as measures of acute response and recovery status.

**Figure 1:**
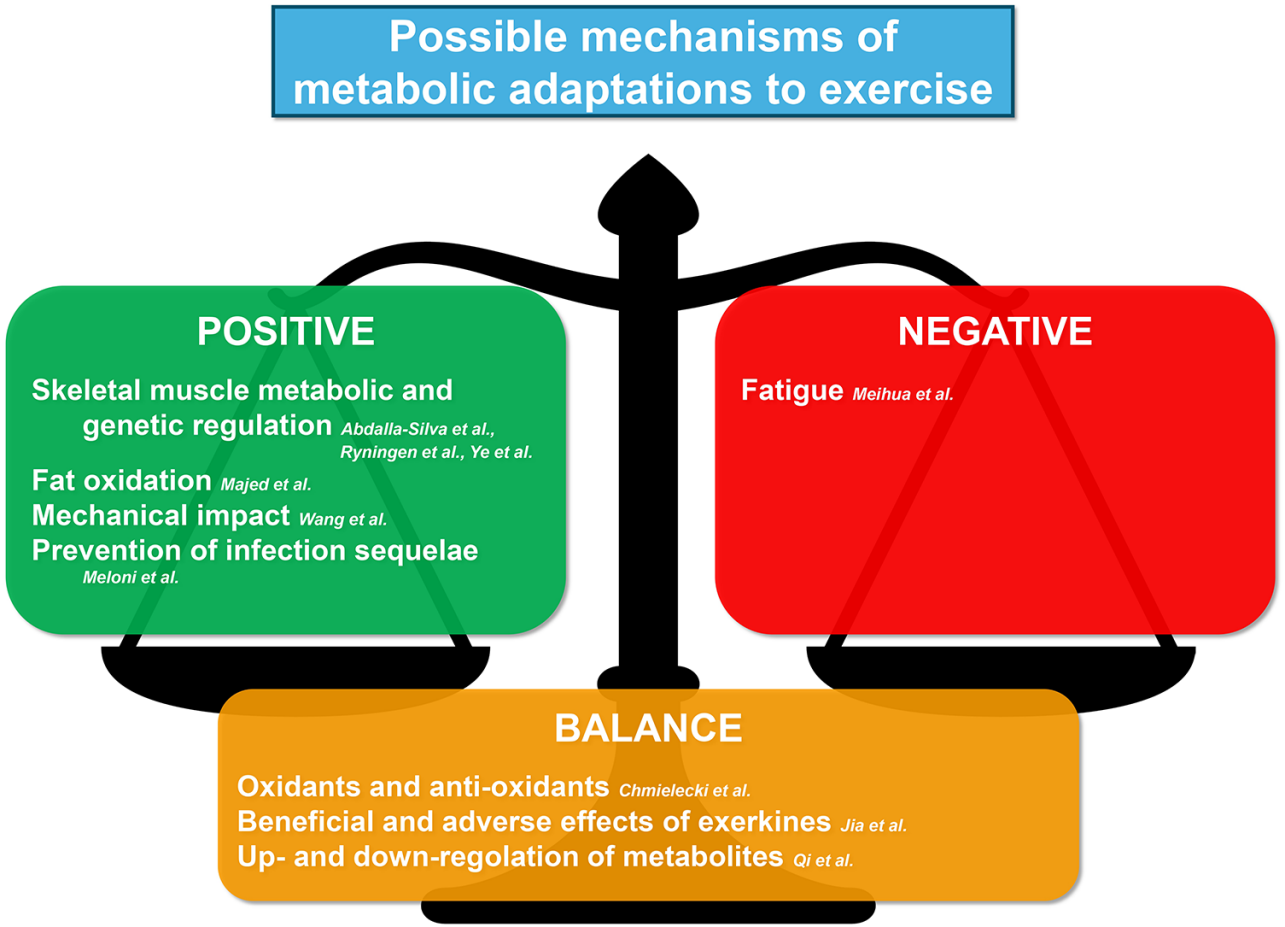
Summary of the contribution of this Research Topic to understanding the possible mechanisms of metabolic adaptations to exercise.

Collectively, the published manuscripts in this Research Topic have contributed meaningful novel findings as summarized in Figure 1, but there are still many unsolved issues in metabolic responses and adaptations to exercise and more research on physical activity effects are needed. The final goal is to advance our mechanistic understanding of exercise metabolism in tissues and systems throughout the body, explore the therapeutic benefits of exercise, and integrate these findings with other emerging themes across the metabolic arena. This knowledge may help to better target interventions preserving health and fitness and managing diseases, as well as to improving performance in athletes.

